# Zimmermann-Laband syndrome: Clinical and cytogenetic study in two related patients

**DOI:** 10.4317/jced.55214

**Published:** 2019-05-01

**Authors:** Sadegh Shirian, Hassan Shahabinejad, Abolfazl Saeedzadeh, Khosrow Daneshbod, Hengameh Khosropanah, Mostafa Mortazavi, Yahya Daneshbod

**Affiliations:** 1 Department of Pathology, School of Veterinary Medicine, Shahrekord University, Shahrekord, Iran; 2 Shiraz Molecular Pathology Research Center, Dr Daneshbod Pathology Laboratory, Shiraz, Iran; 3 Biotechnology Research Inistitute, Shahrekord University, Shahrekord, Iran; 4 Department of Endodontics, Henry M Goldman School of Dental Medicine, Boston University Boston, MA, USA; 5 Department of Periodontology, School of Dentistry, Shiraz University of Medical Science, Shiraz, Iran; 6 Craniomaxillofacial Surgery Research Center, Shariati Hospital, Tehran University of Medical Sciences, Tehran, Iran

## Abstract

**Background:**

Zimmermann–Laband Syndrome (ZLS) is an extremely rare autosomal dominant congenital disorder. It is a craniofacial malformation syndrome with predominant intraoral involvement consisting of gingival fibromatosis diffusion in early development. The molecular basis of ZLS is still unknown. Although familial aggregation with different inheritance patterns is detected in ZLS patients, most of the cases are sporadic.

**Material and Methods:**

We report on two sibling patients with clinical manifestations of ZLS. Blood samples of both patients were obtained in EDTA-tubes followed by performing cytogenetic study using Cyto2.7M array. Analysis of the copy number was performed using the Chromosome Analysis Suite Software (version 1.0.1, annotation file na 30, Affymetrix) and interpreted with recourse to the UCSC genome browser (http://genome.ucsc.edu/; Human Mar. 2006NCBI Build 36.1/hg18 assembly).

**Results:**

The array analysis revealed overlapping regions of chromosomal aberrations in both patients. We detected a 258-kb deletion at 3q13.13, a 89-kb duplication at 1q25.2 as well as two 67-kb duplications at 1p12 and 19q12. These altered regions do not contain any known genes and protein-coding sequences.

**Conclusions:**

In conclusion, the findings of this report revealed new chromosomal aberrations, including a deletion at 3q13.13 and duplications at 1q25.2, 1p12 and 19q12, in the two patients with ZLS. Such findings indicate that whole genome screening for genomic rearrangements is fruitful in typical and atypical patients with ZLS.

** Key words:**Zimmermann-Laband syndrome, cytogenetic array, whole genome screening, chromosomal aberration, gingival fibromatosis.

## Introduction

Zimmermann-Laband syndrome, also known as Laband’s syndrome, is a very rare genetic disorder characterized by gingival fibromatosis (GF), hypo/aplastic nails and distal phalanges, abnormalities of soft cartilages of the nose and/or ears, hepatosplenomegaly, hyperextensibility of joints, mild hirsutism, hypertrichosis and intellectual disability (1). Gingival fibromatosis, which is involved in development of masticatory, esthetic and speech problems, is the most explicit manifestation of ZLS. The characteristics of ZLS are highly variable and complicated. Both females and males equally are affected by this disease. Gingival fibromatosis is usually present at birth or appears shortly after. It is characterized by slow and progressive enlargement of the maxillary and mandibular gingiva (2). Hypo/aplastic nails and facial features are the distinguishing characteristics of ZLS from other cognate disorders (3). Whether these features stand for distinct etiology or rather clinical variability remains unknown. Although, it has been suggested that the inheritance pattern of ZLS is autosomal dominant, its molecular basis is yet to be elucidated. Despite of the familial aggregation with different inheritance patterns found in ZLS patients, most of the cases are sporadic.

Several methods, including FISH, comparative genomic hybridization (aCGH) and whole-genome technology, have been recently applied for detection of common chromosomal aberrations, such as breakpoints, deletions, duplications, insertions and inversions. Whole-genome technology has provided the opportunity to identify the numerous previously unrecognized micro-deletion and micro-duplication syndromes (4).

The present study reports on two sibling children with atypical clinical features of ZLS as well as novel chromosomal anomalies detected using Whole-genome 2.7 M array.

## Material and Methods

**Figure 1:**
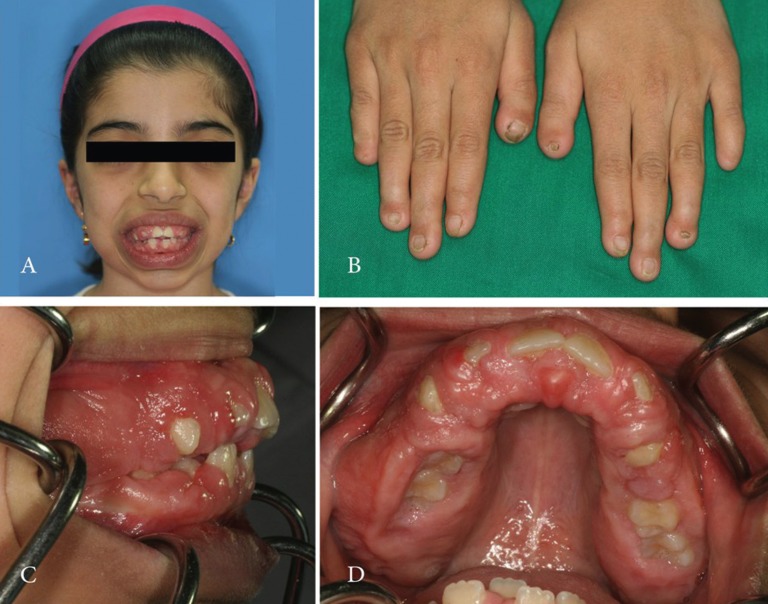
Nine-year-old female patient with a Zimmermann–Laband syndrome phenotype. Note gingival hypertrophy and thick lips (A, C, and D). Nails of all fingers are hypoplastic. Absence of fingernails of the first fingers on the left and right hand is seen (B).

**Figure 2:**
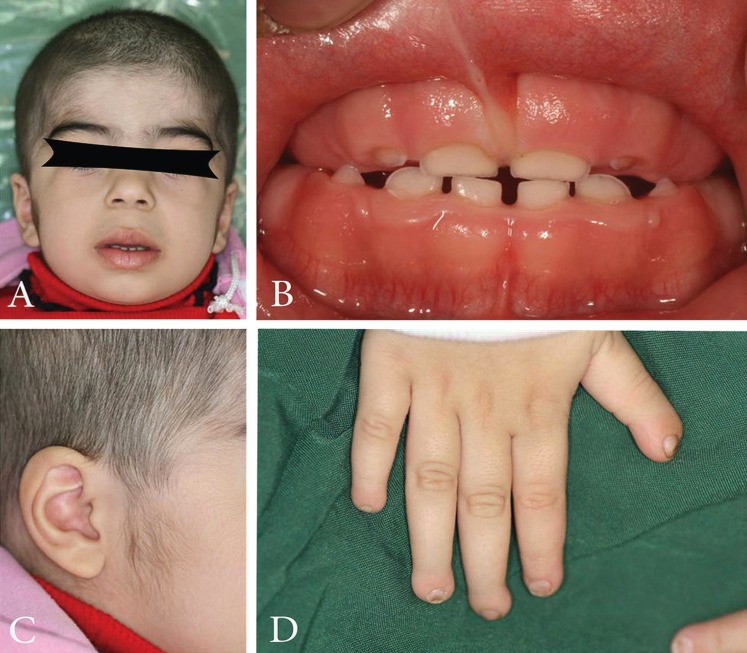
Three-year-old female patient with a Zimmermann–Laband syndrome phenotype. Note hypertrichosis (A and C) and gingival hypertrophy (B). Nails of all fingers are hypoplastic are dystrophic (D).

A 9-year old girl was referred to the oral surgery clinic because of “gum overgrowth”. Prior to her referral, she had undergone surgical procedures, namely rectal polyp, renal sx due to hydrouretronephrosis and adenoid tonsilectomy, when she was one, four and six years old, respectively. Moreover, she had a history of small ventral septal defect (VSD) G>52mmhg, hepatosleenomegaly and growth. Her medical issues were congenital and recurred after three gingival surgeries performed over the past four years. Gingival hypertrophy (Fig. 1A, C, and D), telecanthus, hepatosplenomegaly, hypertrichosis and hypoplasia of terminal phalanges (Fig. 1B) with nail hypoplasia were detected during physical examination. Her appearance was otherwise normal. Microscopic findings revealed dense hypocellular, hypovascular collagenous tissue with numerous bundles in all directions; furthermore, the surface epithelium exhibited long thin rete ridges which were extend deeply into the underlying fibrous connective tissue without any inflammation. Her 3-year old sister was also suffering from gingival and phalangeal hypertrophy (Fig. 2 B and D), in addition to hypertrichosis (Fig. 2A and C). They had no history of epilepsy and did not take any medications, such as anticonvulsants. Their prenatal history, including the medications their mother had taken during her pregnancies, was not significant. Their parents and grandparents were cousins with insignificant medical history. Their other two siblings had no medical issues. Examining the gingival biopsy samples obtained from both patients indicated dense fibroblastic cell proliferation with numerous bundles in all directions in favor of gingival fibromatosis. The clinical manifestations and the pattern of inheritance were consistent with ZLS. Cytogenetic study was performed to detect the possible chromosomal aberrations in the patients. In contrast to the findings of other ZLS-related reports, none of the patients had intellectual disability .

The Ethics Committee of the Faculty of the Dentistry School, University of Shiraz Medical Sciences (Shiraz, Iran) approved the study, and the author group collected written, informed consent from both patients.

## Results

Blood samples were obtained from both patients in EDTA-and sent to the Institut für Humangenetik, UNIVERSITÄTSKLINIKUM Schleswig-Holstein, Germany. DNA-extraction for Cyto2.7M array (Affymetrix, Santa Clara, CA, USA) was performed according to the protocols provided by the manufacturer (Affymetrix, Santa Clara, CA) (http://www.affymetrix.com), Microarrays were washed and stained with the Fluidics Station 450 (Affymetrix, Santa Clara, CA) and scanned using the Gene Chip Scanner 3000 (Affymetrix, Santa Clara, CA). Copy number analyses of the Cyto2.7M array was performed using the Chromosome Analysis Suite Software (version 1.0.1, annotation file na 30, Affymetrix) and the data were interpreted with the aid of the UCSC genome browser (http://genome.ucsc.edu/; Human Mar. 2006NCBI Build 36.1/hg18 assembly). The detected genome regions, that were marked as significant by the software, were considered to be aberrant if more than 50 altered markers were included within a window of more than 50kb. The distinction between pathological aberrations and copy number variations (CNV) was performed using the Database of Genomic Variants implemented in the software.

Analyses of the arrays revealed overlapping regions of chromosomal aberrations in both patients. We detected a 258-kb deletion of at 3q13.13 (hg18 coordinates chr3: 111.204.741-111.463.070), a 89-kb duplication at 1q25.2 (hg18 coordinates chr1: 175.712.702-175802.226) as well as two 67-kb duplications at 1p12 (hg18 coordinates chr1: 118.948.407-119.016.322) and 19q12 (hg18 coordinates chr19: 33.899.107-33.966.140). These altered regions did not contain any known genes and protein-coding sequences.

## Discussion

Patients with genomic instability disorders generally display recognizable patterns of manifestations that lead to a clinical diagnosis (5). ZLS is an extremely rare autosomal dominant congenital disorder. It is a craniofacial malformation syndrome with predominant intraoral involvement consisting of gingival fibromatosis diffusion in early development (6). Other features of ZLS include large protruding upper lip, large facial bones and mandibles, strabismus, as well as enlarged phallus, fingers and toes (7). The differential diagnoses of GF consist of pregnancy, inflammation, leukemia and exposure to drugs, such as phenytoin, diltiazem, cyclosporine A, verapamil, and nifedipine (1). Gingival fibromatosis, which is the most common characteristic of ZLS, is usually present with hypertrichosis (8). The clinical examination revealed that both patients suffered from gingival and phalangeal problems. Although, the results of the review study conducted by Castori *et al.* (2013) indicated that the combination of gingival hypertrophy and hypo/aplasia of the nails, with or without phalangeal involvement, are the most explicit characteristics of ZLS and are present in all (100%) patients (3), hypo/aplasia of the nails was not detected in one our patients. Both patients suffered from hypertrichosis, however, one of them had hepatosplenomegaly. Other commonly reported features ZLS include joint hypermobility, hypertrichosis and hepatomegaly with or without splenomegaly (3).

The molecular basis of ZLS is still unknown. In 2003, Stefanova *et al.* have described an apparently balanced reciprocal t (3;8) (p21.2;q24.3) translocation in a mother and daughter with ZLS (9). Moreover, an apparently balanced de novo t (3;17) (p14.3;q24.3) translocation found in a boy with ZLS has been reported by Kim *et al.* (2007). A chromosal breakpoint of 200-kb region was detected on chromosome 3p14.3 by FISH analysis (7). Kim *et al.* (2007) have reassessed the chromosome 3p breakpoint described by Stefanova *et al.* (2003) and revised the breakpoint location to a 3.2-Mb region in chromosome 3p14.3, which includes the CACNA2D3 and WNT5A genes. According to these results, they have suggested that the gene responsible for ZLS is located at 3p14.3. However, Hoogendijk, *et al.* (2006) have reported a new chromosomal insertion, ins (12;8)(p11.2;q11.2q24.3), in a patient diagnosed with ZLS. They have concluded that the gene responsible for the syndrome lies on chromosome 8 (10). In 2013, Castori et al. have studied two unrelated children with full-blown characteristics of ZLS and excluded chromosome imbalances at aCGH resolution of 75 Kb in both patients (3). In our study, no aberrancies were identified at the 3p14.3 which have previously been found in patients with the typical clinical features of ZLS. None of these genes were directly implicated in any of the phenotypes of our patient, or has any patient been described with this specific chromosomal aberrations. We detected a deletion at 3q13.13 and duplications at 1q25.2, 1p12 as well as 19q12.

Congenital deletions affecting 1q have rarely been reported (11). Facial dysmorphology as well asconstant flexion of the proximal interphalangeal joints, and short distal phalanges and nails of fingers are correlated with an additional band in 1q (12). Duplication defects of chromosome 1 are also rare. A newborn male with duplication in chromosome 1p.34 and multiple congenital abnormalities has been reported by Warden *et al.* (13). Our cytogenetical analyses revealed duplications at 1q25.2 and 1p12 regions of both patients. Therefore, we suggest that not only the congenital deletions of 1q that have previously been reported as causative facial and phalangeal dysmorphology but also any chromosomal aberration at this region, such as duplication, can result in these anomalies. In our previous review article, we have not found any cases with the same duplication as that of the patients of this patient. The abnormalities detected in this study suggest that this region of chromosome 1p and 1q could be involved in determining cell migration and/or differentiation during organogenesis.

A 67-kb duplication was detected in chromosome 19q12 of both patients. To the best of our knowledge, this is the first report of ZLS patients with duplication in 19q12. It is possible that this duplication is resulted from defective ATM-dependent DNA double strand break repair (14), which is an active process during meiosis (15) as well as mitotic cell cycling, and may underlie the recently described phenomenon of “chromothripsis”. However, it is not clear whether the de novo duplication in 19q12 occurred during the paternal or maternal meiosis, or during an early postzygotic cell division (16).

In conclusion, the findings of the present study revealed new chromosomal aberrations, including a deletion at 3q13.13 as well as duplications at 1q25.2, 1p12 and 19q12, in the two sibling patients with ZLS. These findings indicate that whole genome screening for genomic rearrangements is useful in both typical and atypical patients with ZLS.
